# Microstructure and pore structure of polymer-cement composite joint sealants

**DOI:** 10.1038/s41598-021-81088-9

**Published:** 2021-01-14

**Authors:** Chuanxin Lou, Jinyu Xu, Tengjiao Wang, Weibo Ren

**Affiliations:** 1 Aeronautics Engineering College, Air Force Engineering University, Xi’an, 710038 China; 2 College of Mechanics and Civil Architecture, Northwest Polytechnic University, Xi’an, 710072 China

**Keywords:** Engineering, Materials science

## Abstract

Utilizing methods such as scanning electron microscopy observation and mercury intrusion porosimetry, this paper investigates the basic microstructure and pore structure properties of polymer-cement composite joint sealants for pavements, and analyzes the effects and rules of various material types, ratio parameters and processing conditions. Further, the fractal characteristics and variation rules of pore size distribution are investigated for the joint sealants by introducing the fractal theory. The results show that changes in material type, ratio parameter and processing condition produce insignificant effects on the basic microstructure properties and configuration of joint sealants, with effects reflected primarily in the change of sealant pore structure. Measures like increasing the powder-liquid ratio and cement ratio, blending with sulphoaluminate cement or mica powder, adding latex powder or coupling agent, cold drawing and hot pressing, as well as ultraviolet irradiation treatment are all capable of reducing the total pore volume of joint sealants and refining their pore structure. In contrast, opposite effects are yielded when low-grade cement is used, styrene-acrylic emulsion is blended, or plasticizer is added. Additionally, after blending with talc powder or adding carbon fiber additive, the total pore volume of joint sealants remains basically unchanged or reduced, despite the coarsened pore structure. The total pore volume of joint sealants increases after wet–dry cycling treatment, while no obvious change in the pore size distribution is observed. Pore size distribution of the studied joint sealants presents distinct fractal characteristics, and the corresponding fractal dimension of pore surface area ranges between 2.6 and 2.8.

## Introduction

Polymer-cement composite is a novel type of joint sealant based on the “organic–inorganic composite” concept.The polymer-cement composite can have advantages of both inorganic materials and organic materials, such as high durability, high strength, and excellent deformation^1–3^.

Forming of polymer-cement composites mainly comprises the hydration and hardening process of cement fraction, the dehydration and film forming process of polymer fraction, as well as the reaction between cement hydrate and polymer fraction^4,5^. Accordingly, the most remarkable feature of such materials at the microscopic level is a three-dimensional hybrid structure, which is formed by the interweaving of cement hydrate and polymer film structure^6^. Given the different material types, ratio parameters and fabrication technologies, the hybrid structures have complicated microstructure and material composition, which in turn diversifies the mechanics and durability of the composites at the macroscopic level, thus allowing the composites to have technical performance advantages over many other simple cement-based or polymer-based materials. This is precisely the basic idea and principle of polymer-cement blending modification. Hence, microscopic research on the morphology, structure, composition, etc. of polymer-cement composites is helpful for understanding the interaction mechanism between polymers and cements, which thereby facilitates the fundamental analysis and explanation of different property characteristics reflected by various polymer-cement composites. Itprovides a scientific basis for improving the utilization rate and application effects of polymers, for cutting their application cost, and for further optimizing the material design.

The research topics concerning the microstructure and phase changes of polymer-cement composites include the microstructure properties, pore structure distribution, material composition changes, various physicochemical reactions, and correlations between microstructure and macroscopic properties of the materials, where the influencing factors involved are material type, ratio parameter, curing age, pretreatment method, etc.^7^. Su et al.^8^ studied the microstructure evolution process of styrene-acrylic emulsion-modified cement over different early periods. By means of SEM, DTA and MIP, Ollitrault-Fichet et al.^9^ explored the effects of polyacrylate emulsion on the microstructure, material composition and pore structure of cement mortars. According to a study on the mechanical properties of various polymer-modified mortars by Beeldens et al.^10^, the density, porosity and location of film structure were correlated with the type of polymer material used, as well as the minimum film forming temperature. In a SEM-based study by Afridi et al.^11^, the coalescence morphologies of polymer particles in the polymer powder- and emulsion-modified mortars were analyzed. On the basis of investigating the Vickers hardness, pore structure and polymer-cement interaction of SBR emulsion-modified mortar, Rozenbaum et al.^12^ proposed a Monte Carlo model for characterizing the correlation between microstructure of the material and its macro-mechanical properties. Yu et al.^13^ analyzed the correlations of microstructure of JS waterproof coating film with its tensile properties and water resistance based on the SEM results. Qiao et al.^14^ observed the microstructure properties of redispersible latex powder-modified mortar, including the cement hydration product, polymer film structure, interfacial transition zone, etc. Furthermore, they tested the pore structure distribution of the modified mortar, and analyzed the mechanism whereby the polymer emulsion powder improves the mortar mechanical durability from the microscopic perspective. Ren et al.^15^ explored the microstructure and pore structure of sulphoaluminate cement mortars that were modified with styrene-acrylic and pure acrylic emulsions over various ages. By a combination of FTIR, XPS and GPC, Wang et al.^16^ systematically explored the chemical reaction mechanisms in polyacrylate emulsion-modified cement materials.Ma et al.^17^ probed into the fractal characteristics of calcium-based polymers, and discussed the correlations between fractal dimension, pore structure parameters and macro-mechanical properties. Havlin et al.^18^ found thatthe fractal dimensionality was well defined and had a constant value for most scales of length of the chain. Konkol^19^ demonstrated the connection between the fractal dimension and the investigated properties of concrete. Extant studies concerning polymer-cement composites are dominated by rigid materials with low polymer-cement ratios, while flexible composite materials with high polymer-cement ratios are scarcely investigated. The systematic studies exploring the effects of changes in material type, ratio parameter and processing condition on the microstructure and pore structure of polymer-cement composite joint sealants are even rarer.

This paper studies the basic microstructure and pore structure properties of polymer-cement composite joint sealants with high polymer-cement ratios mainly by SEM observation and MIP, and analyzes the effects and rules of various material types, ratio parameters and processing conditions. Then, further investigation is made into the fractal characteristics and variation rules of pore size distribution for the joint sealants by introducing the fractal theory, thereby revealing the effects of changes in material type, ratio parameter and processing condition on the sealants at the microscopic level. The research results are conducive to fundamentally understanding the interaction mechanism between organic and inorganic fractions inside the materials, as well as the effects and mechanisms of various factors on their macroscopic properties. Thus, they provide a scientific basis and a theoretical support for the performance evaluation, failure analysis and optimal modification of the polymer-cement composite joint sealants.

## Experimental details

### Materials

Depending on property and function, the raw materials used in the preparation of polymer-cement composite joint sealants can be divided into three major parts: Base materials, filler materials and additives. Among them, the base materials include various polymer emulsions and cements. Regarding selection of emulsions, the Acronal S400F styrene-acrylic ester copolymer emulsion (i.e. styrene-acrylic emulsion) (BASF, Germany) and the Celvolit 1350 vinyl acetate-ethylene copolymer emulsion (i.e. VAE emulsion) (Celanese, USA) are used as the raw materials of polymer emulsions. As for cements, the "Yaobai" brand grade 42.5 ordinary Portland cement (P∙O 42.5), the grade 32.5 white cement (P∙W 32.5) and the grade 42.5 rapid-hardening sulphoaluminate cement (R∙SAC 42.5) (Yaobai Cement Co., Ltd., Shaanxi, China) are used. Filler materials are various inorganic mineral powders, including talc powder, quartz powder and mica powder. Additives are mainly various functional admixtures that assist in the improvement of polymer film formation, cement hydration, powder dispersion, etc. Specifically,the redispersible latex powder used is the 5044 N VAE copolymer powder (Wacker, Germany); the dispersant, defoamer and coalescing agent used are the SN-5040 dispersant (San Nopco, Japan), SN-345 defoamer (San Nopco, Japan) and DN-12 coalescing agent (Tianyin Chemical Co. Ltd., Jiangsu, China) respectively; the plasticizer used is the dioctyl phthalate with a purity of ≥ 99%(Zhiyuan Chemical Reagent Co., Ltd., Tianjin, China); the coupling agent used is the KH-550 silane coupling agent (Yingchu Chemical Technology Co., Ltd., Jinan, China); the retarder used is the HWR Q018 powder retarder (Qinfen Building Materials Co., Ltd., Shaanxi, China); and the fiber reinforced material used is the AKSACA chopped carbon fiber with a tensile strength of 4100 MPa (Turkey).

### Specimen preparation

**Figure 1:**
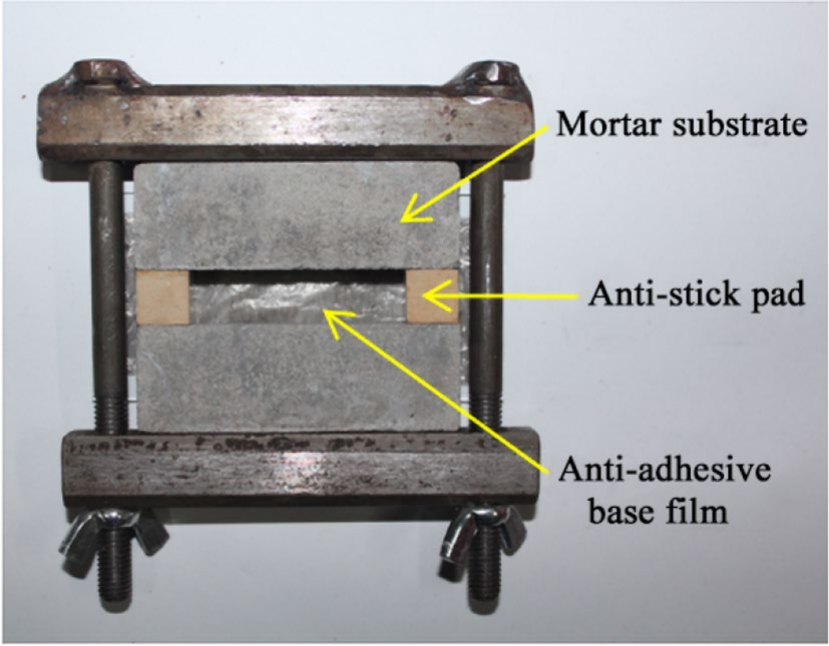
Mold.

**Figure 2:**
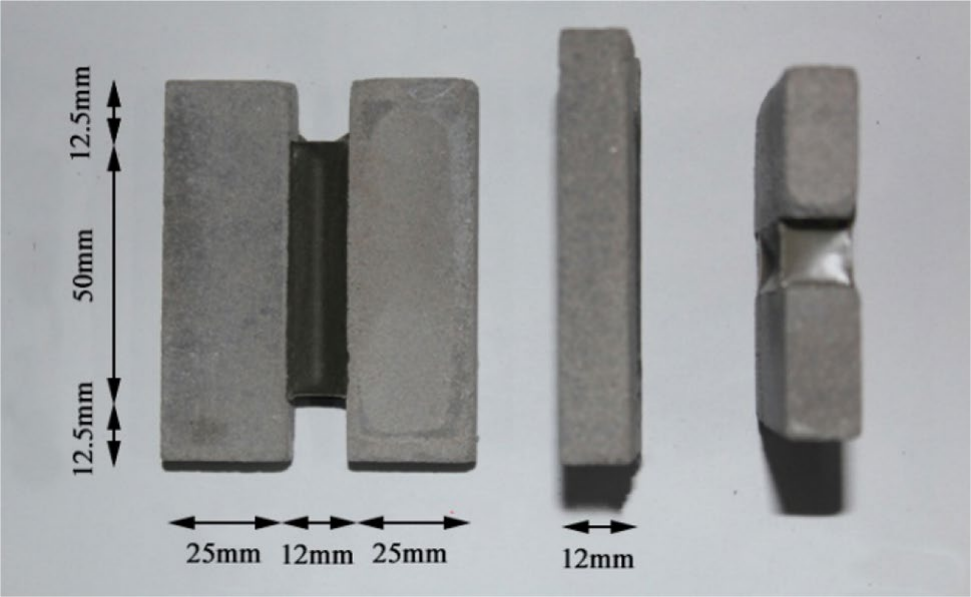
Specimen.

The test specimens are prepared in a total of six steps, namely material weighing, additive dispersion, powder dispersion, low-speed stirring defoaming, pouring and curing. Specific details are as follows: (1) The raw materials of various components are weighed according to the mix proportions shown in Table 1. (2) The dispersant, coalescing agent and half of defoamer are incorporated into the emulsion, and then stirred for 150 s using a high-speed electric mixer at a rotation speed of 300 r/min (measurement and adjustment of the rotation speed are accomplished by photoelectric tachometer). (3) After dry mixing and uniform stirring, all powder materials are incorporated into the emulsion (in a stirring state), and then stirred for 10 min at a 700 r/min high speed to ensure no powder agglomeration is observed. (4) The remaining half of defoamer is then incorporated, which is stirred at 120 r/min for 3 min initially, followed by manual low-speed stirring with a stirring rod for 10 min, in order to ensure absence of apparent air bubbles in the mixture. (5) Using a glue injector, the well-mixed mixture is injected into a cavity comprising cement mortar base, anti-adhesive pad and anti-adhesive bottom film (see Fig. 1), where the base material and pad dimensions are set. (6) The cast specimens (see Fig. 2) are placed in the indoor environment, and removed of the anti-stick pad and anti-adhesive base film after curing for 4d. Then, the curing is continued for an additional 24d.

**Table 1:** Mix proportion.

Specimen No	powder-liquid ratio	Ceme% nt ratio (%)	VAE emulsion	Styrene-acrylic emulsion	Quartz powder	Talc powder	Mica powder	P·O 42.5 cement	R·SAC 42.5 cement	P·O 32.5 cement	Redispersible latex powder	Plasticizer	Coupling agent	Carbon fiber	Dispersant	Defoamer	Coalescing agent
DZ	0.45	35	100	–	29.2	–	–	15.8	–	–	–	–	–	–	1.02	0.73	6
Y1	0.30	35	100	–	19.5	–	–	10.5	–	–	–	–	–	–	1.02	0.73	6
Y2	0.55	35	100	–	35.8	–	–	19.2	–	–	–	–	–	–	1.02	0.73	6
CR1	0.45	25	100	–	33.8	–	–	11.2	–	–	–	–	–	–	1.02	0.73	6
CR2	0.45	50	100	–	22.5	–	–	22.5	–	–	–	–	–	–	1.02	0.73	6
CT1	0.45	35	100	–	29.2	–	–	–	–	15.8	–	–	–	–	1.02	0.73	6
CT2	0.45	35	100	–	29.2	–	–	11.1	4.7	–	–	–	–	–	1.02	0.73	6
T1	0.45	35	100	–	17.5	11.7	–	–	–	15.8	–	–	–	–	1.02	0.73	6
T2	0.45	35	100	–	17.5	–	11.7	–	–	15.8	–	–	–	–	1.02	0.73	6
H	0.45	35	80	20	29.2	–	–	15.8	–	–	–	–	–	–	1.02	0.73	6
J	0.45	35	100	–	29.2	–	–	15.8	–	–	5.0	–	–	–	1.02	0.73	6
Z	0.45	35	100	–	29.2	–	–	15.8	–	–	–	2.0	–	–	1.02	0.73	6
L	0.45	35	100	–	29.2	–	–	15.8	–	–	–	–	0.90	–	1.02	0.73	6
X	0.45	35	100	–	29.2	–	–	15.8	–	–	–	–	–	0.145	1.02	0.73	6
K0	0.40	40	100	–	14.4	9.6	–	16	–	–	–	–	–	–	0.98	0.70	6
K1	0.40	40	100	–	14.4	9.6	–	16	–	–	–	–	–	–	0.98	0.70	6
K2	0.40	40	100	–	14.4	9.6	–	16	–	–	–	–	–	–	0.98	0.70	6
K3	0.40	40	100	–	14.4	9.6	–	16	–	–	–	–	–	–	0.98	0.70	6

**Figure 3:**
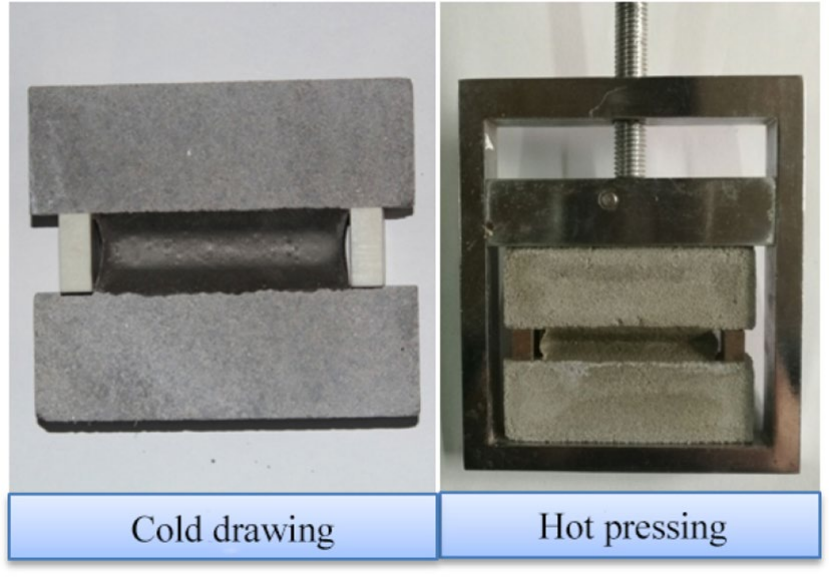
Cold drawing and hot pressing.

Pretreatment is carried out by consulting the methods in GB/T 13,477^20^. The specimens in groups K1, K2 and K3 are pretreated as follows:

Cold drawing and hot pressing: The specimens in group K1 are stretched to a specified width (25% of the original specimen width) and kept at -20℃ for 24 h. Then, the stretching is terminated, and the specimens are compressed to a specified width (25% of the original specimen width) and kept at 70℃ for 24 h, as shown in Fig. 3. The compression is discontinued after repeating this treatment six times.

Wet–dry cycling treatment: Initially, the specimens in group K2 are soaked at water for 2 d, and then taken out and dried naturally at room temperature for 2 d. After repeating this process ten times, the specimens are let stand at room temperature for an additional 7 d, in order to ensure the fundamental evaporation of the internal moisture.

**Figure 4:**
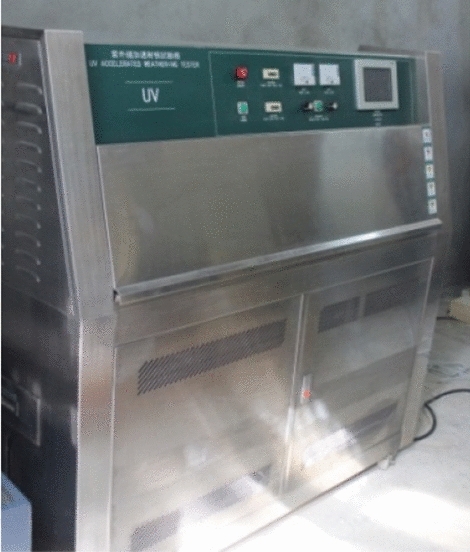
Ultraviolet accelerated weathering tester.

Ultraviolet irradiation treatment: During the experiment, the specimens in group K3 are moved into an ultraviolet accelerated weathering tester(see Fig. 4)for 14 d of ultraviolet irradiation. After completion of the irradiation, the specimens are taken out and let stand at room temperature for 24 h.

In this paper, specimens with different mix proportions of joint sealants are prepared for studying the influence of different factors on the microstructure and pore structure of joint filler, as shown in Table 2.

**Table 2:** Experimental objectives.

Specimen No	DZ	Y1	Y2	CR1	CR2	CT1	CT2	T1	T2
Research objective	Control group	Study the effect of powder-liquid ratio		Study the effect of cement ratio		Study the effect of cement type		Study the effect of filler type	
Specimen No	H	J	Z	L	X	K0	K1	K2	K3
Research objective	Study the effect of styrene-acrylic emulsion	Study the effect of latex powder	Study the effect of plasticizer	Study the effect of coupling agent	Study the effect of carbon fiber	Control group	Study the effect of cold drawing and hot pressing	Study the effect of wet–dry cycling	Study the effect of ultraviolet irradiation

### Test methods

**Figure 5:**
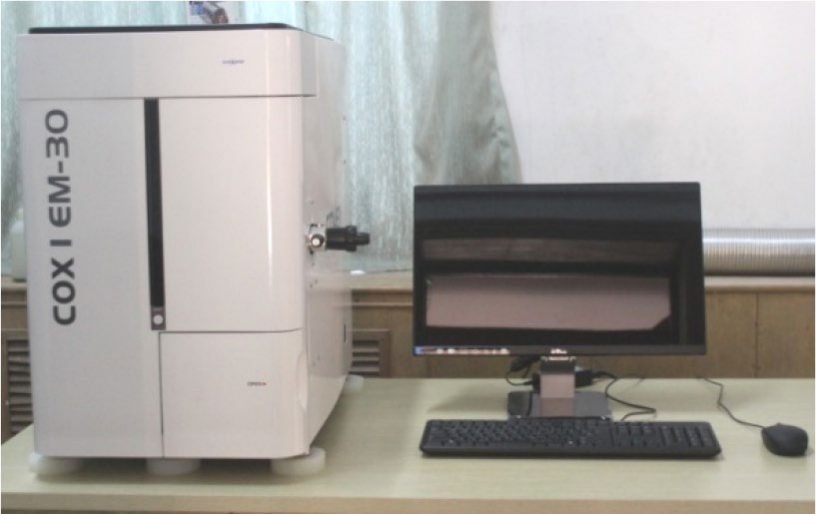
Scanning electron microscope.

**Figure 6:**
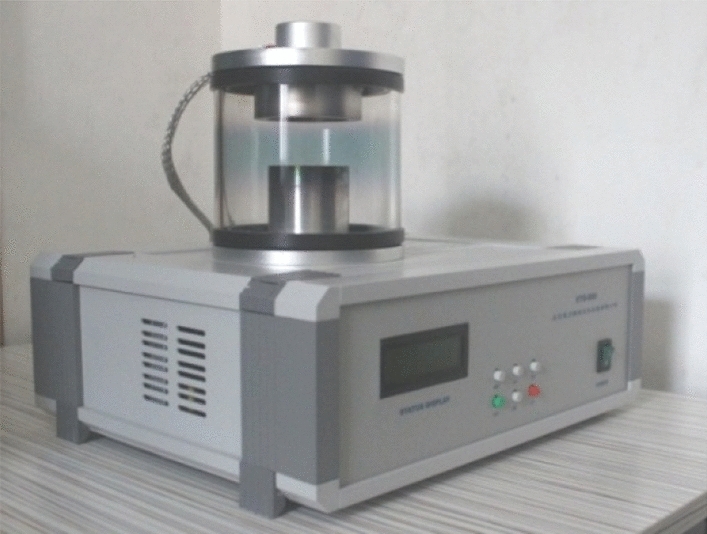
Sputter coater.

The EM-30 scanning electron microscope ( COXEM, South Korea) (see Fig. 5) is used for microstructure observation. Prior to the experimentation, the newly cut specimen sections are sputtered with gold for 90 s using an ETD-800 sputter coater (Woshide Technology Co., Ltd., Beijing) (see Fig. 6), in order to improve the conductivity of the specimen surfaces. Afterwards, the specimens are observed at different magnifications.

**Figure 7:**
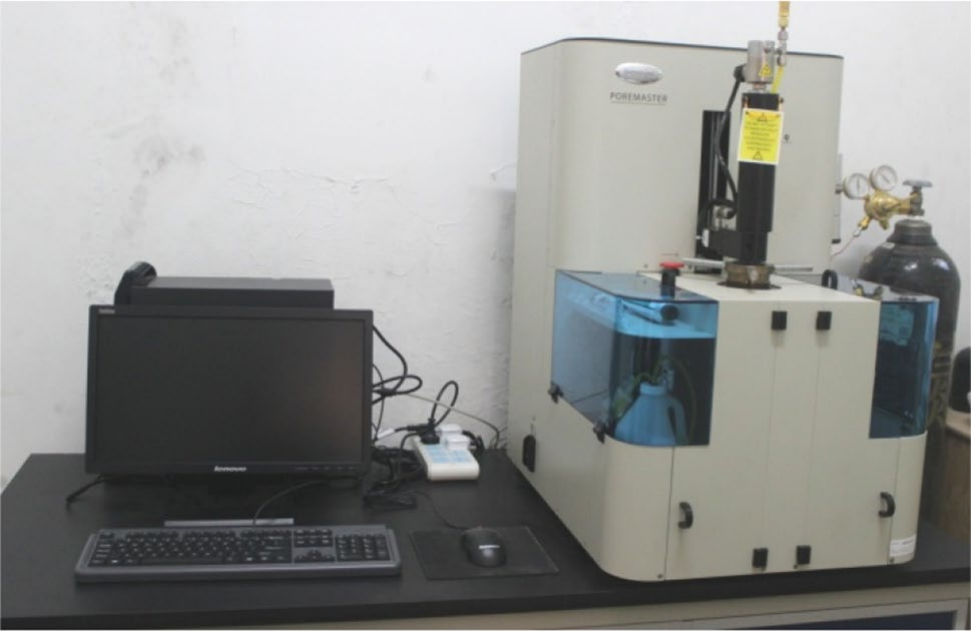
Automatic mercury porosimeter.

Pore structures are determined with a PoreMaster33 automatic mercury porosimeter (Quantachrome Instruments, USA) (see Fig. 7). Prior to the experimentation, the specimen masses are weighed on a precision electronic balance (accuracy 0.1 mg).Then, the specimens are placed separately in the low and high pressure stations for MIP analysis, where the pressure range is set at 20–30,000 psi, and the mercury contact angle is 140°.

## Analysis of microstructure properties

**Figure 8:**
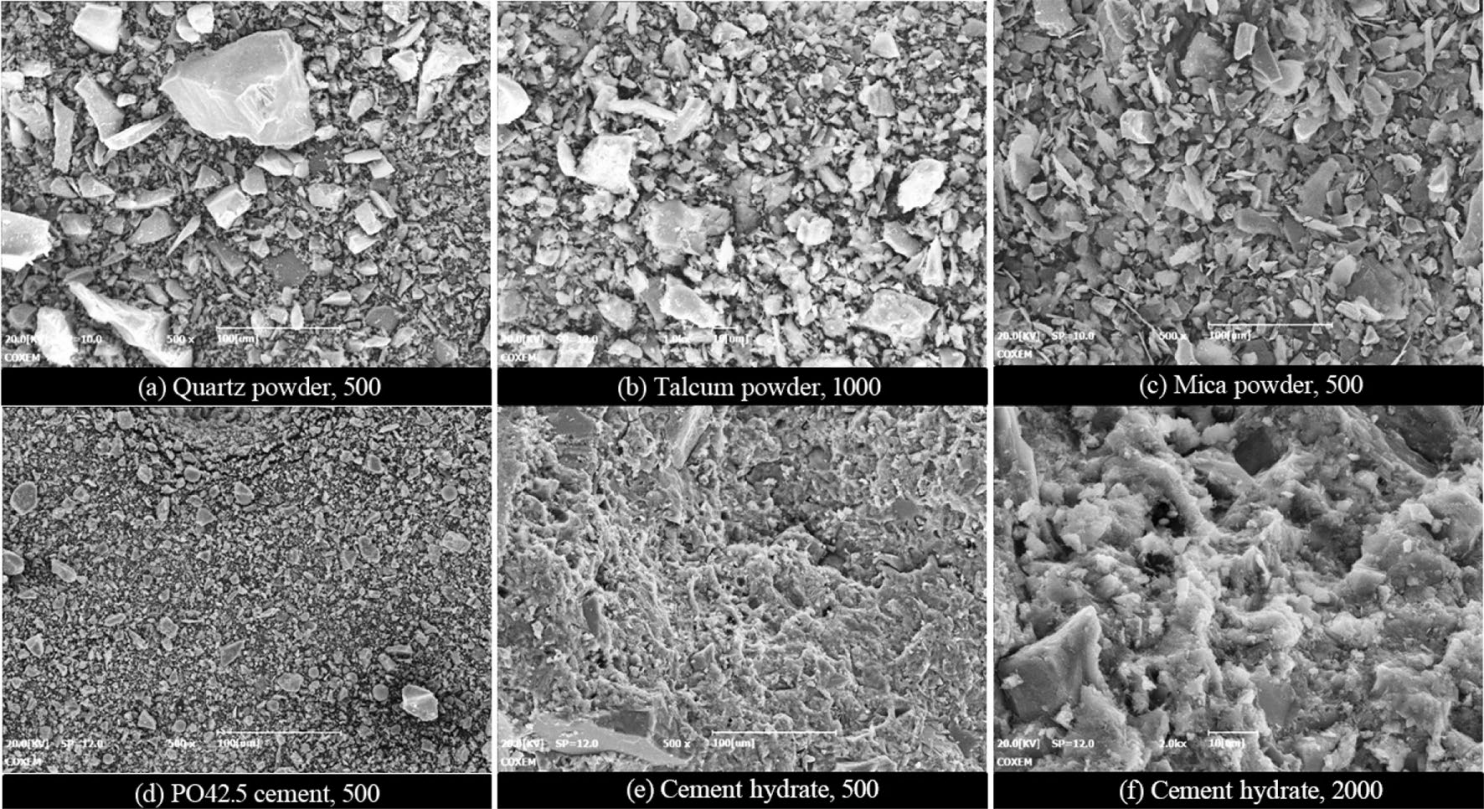
Microstructure of inorganic powder particles and cement hydrates.

**Figure 9:**
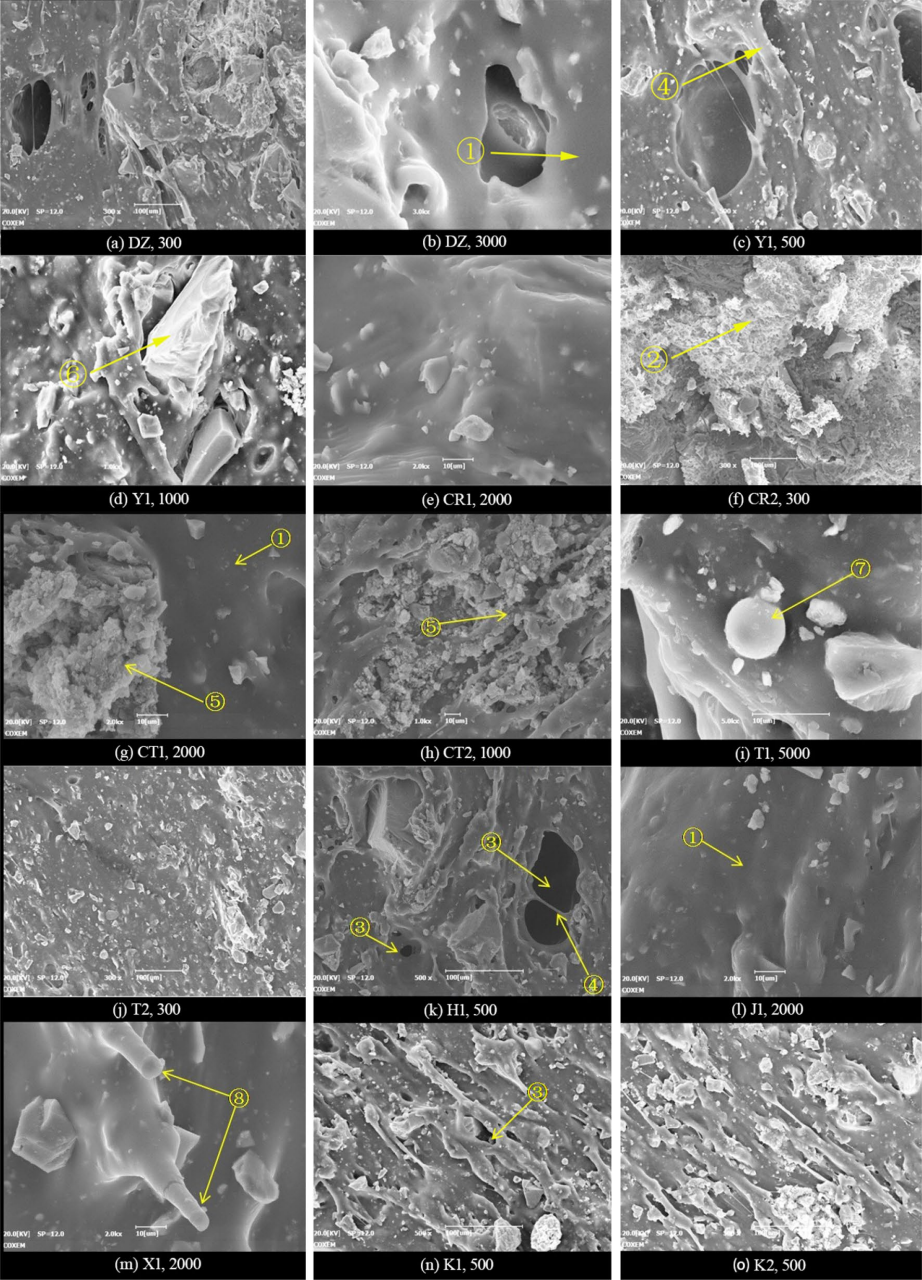
Typical microstructure of polymer-cement composite joint sealant. ①Polymer film; ②Interpenetrating network formed by polymer and inorganic fractions; ③Pores; ④Polymer film transversely penetrating pores; ⑤ Aggregates formed by filler materials and cement hydrates; ⑥Wrapped inorganic fraction; ⑦Unhydrated cement particles; ⑧Wrapped carbon fibers.

In Fig. 8, the micro-morphologies of various involved major inorganic powder particles are presented at different magnifications, as well as the microstructures of P∙O 42.5 cement hydrates. The typical microstructures of joint sealant specimens in various groups are displayed at different magnifications in Fig. 9. As is clear, various inorganic components are embedded and dispersed in the polymer film of the prepared polymer-cement composite joint sealants, which serves as a continuous base phase. Besides, integrative network structure is observable at local sites with relatively enriched inorganic components, which is formed by the interweaving of polymer film with inorganic fillers and cement hydrates. According to Fig. 9, the following microstructure features are further observable:Plentiful pores of different sizes are distributed randomly inside the joint sealants, which are primarily dry shrinkage pores that are formed following water evaporation. Meanwhile, the air bubbles introduced during dispersion and stirring by various gas reaction products can also lead to the generation of certain amounts of pores.Inorganic powders fail to reach an absolutely uniform dispersion state inside the joint sealants, which present an "agglomeration" phenomenon within a certain scale range. Meanwhile, since the fillers and cements are subjected to dry mixing and stirring prior to incorporation of liquid material, the inorganic components like fillers and cement hydrates often accumulate and aggregate with each other after the hardening of sealants. Unhydrated cement particles are observable inside the joint sealants, which are mainly in the form of filler materials. These cement particles may undergo secondary hydration reaction in case the ambient temperature and humidity satisfy relevant conditions.

**Figure 10:**
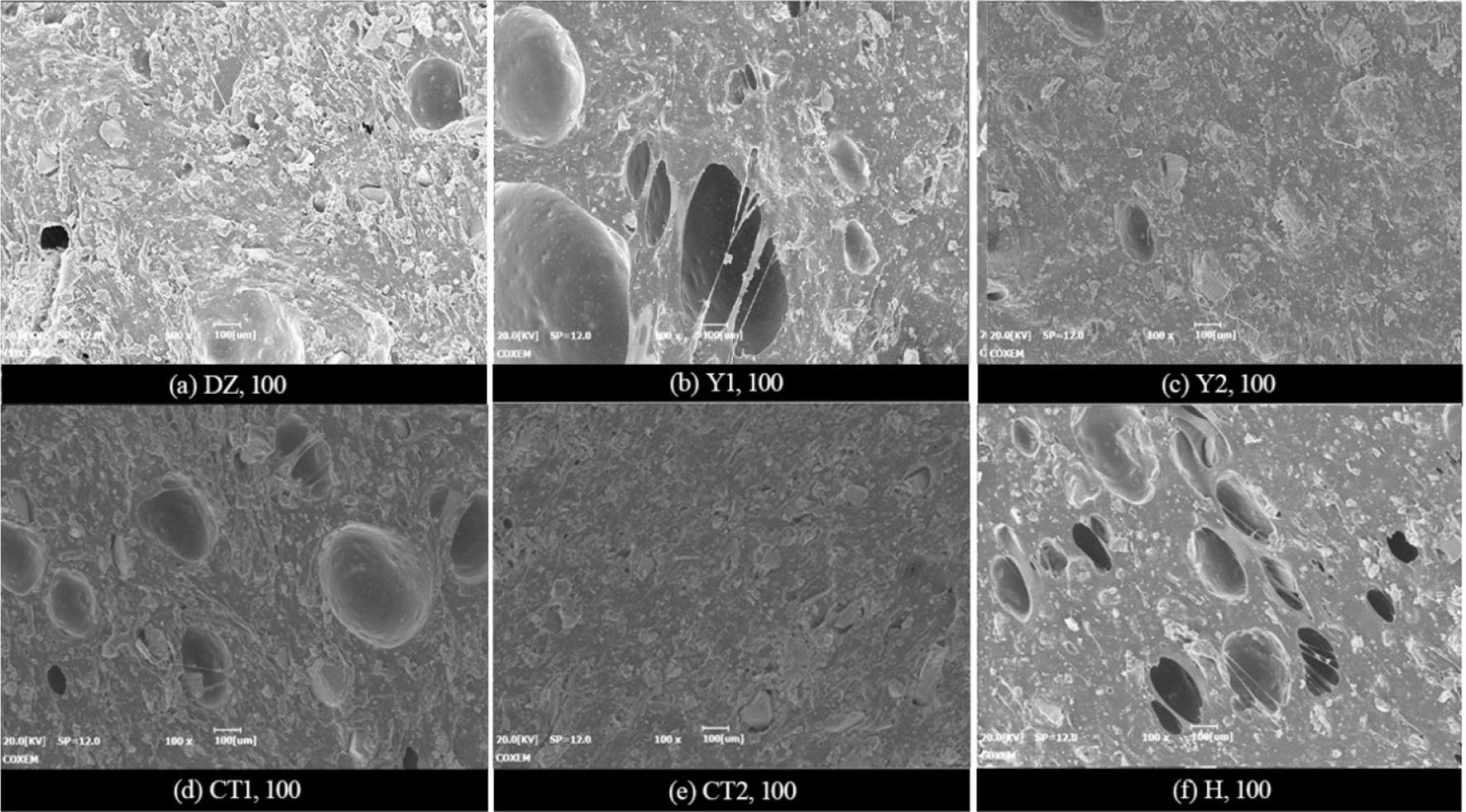
Microscopic pore observation.

According to the micro-morphology comparisons among all specimens in Fig. 9, the overall microstructure differs insignificantly among these specimens, all of which exhibit the aforementioned features. This suggests that the variations of ratio parameters and processing conditions involved do not change the basic microstructure of the joint sealants. Nevertheless, in case of an apparent change in the pore structure of joint sealants resulting from change of individual ratio parameter or processing condition, the corresponding observed size and number of pores will vary markedly. For instance, Fig. 10 displays the low-magnification microstructures of specimens in various groups, which have larger differences in terms of pore observation results. As can be seen, the observed pore size and density values are higher than the control group (DZ),which are achieved by lowering the powder-liquid ratio (Y1), blending with the styrene-acrylic emulsion (H)), or changing to low-grade white cement (CT1). In contrast, the observed pore size and density decrease when the powder-liquid ratio increases (Y2), or a certain quantity of sulphoaluminate cement is blended (CT2). Noteworthy is that in case of changes in the remaining ratio parameters or processing conditions, the pore structures of joint sealants also undergo corresponding changes (as indicated in the analysis in the next section).Nevertheless, this can hardly be reflected in a clear manner through the electron microscope observation.

## Analysis of pore structure properties

### Basic properties of pore structures

Pore size distribution of the polymer-cement composite joint sealants is closely associated with their macro-mechanical properties^21^, and with such durability indicators as anti-permeability and corrosion resistance. Based on the MIP results, this section analyzes the basic pore structure properties of the joint sealants by using the differential curves for pore size distribution, as well as the pore structure parameters. These parameters include the mean pore size (the ratio of total pore volume to the mean pore surface area), the most probable pore size (the pore size corresponding to the peak on the differential curve for pore size distribution), the median pore size (the pore size corresponding to a cumulative mercury intrusion volume of 50%), and the total pore volume.All the pore sizes mentioned in this section refer to the pore diameters.

**Figure 11:**
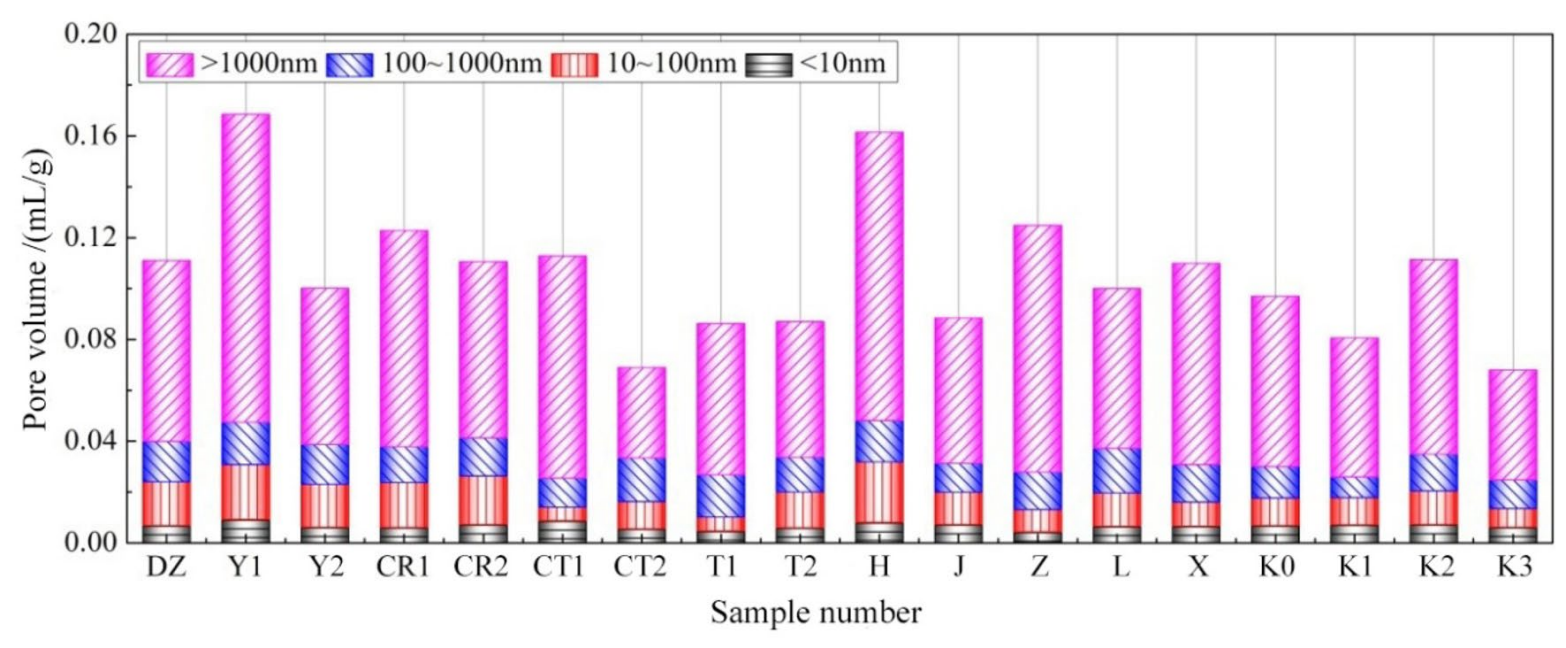
Pore volume distribution.

**Figure 12:**
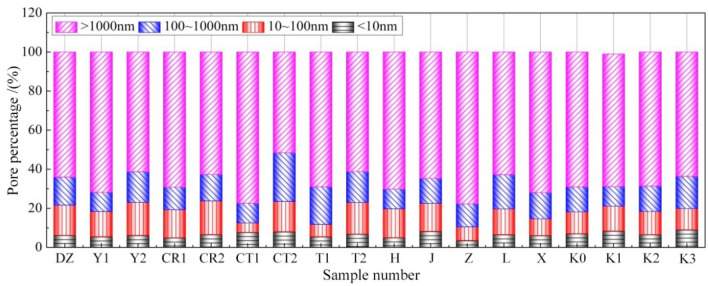
Pore percentage distribution.

In Table 3, the pore structure parameters of tested specimens in various groups are listed. Depending on the pore size, the pore structures measured by MIP in reference^22^ are classified into four types: Macropores (greater than 1000 nm), capillary pores (100–1000 nm), transition pores (10–100 nm), and gel pores (less than 10 nm). In accordance with such classification method, the respective pore volumes of aforementioned four pore types in the present specimens are depicted as shown in Fig. 11, and their percentages in total pore volumes are illustrated in Fig. 12.

**Table 3:** Pore structure parameters.

Specimen No	Mean pore size /nm	Most probable pore size /nm	Median pore size /nm	Total pore volume/(mL/g)	Pore percentage /%				Fractal dimension *D*_p_
					Gel pores < 10 nm	Transition pores 10 ~ 100 nm	Capillary pores 100 ~ 1000 nm	Macropores > 1000 nm	
DZ	63.16	7857	2654	0.1111	6.12	15.48	14.31	64.09	2.7801
Y1	72.86	9261	5092	0.1685	5.46	12.82	9.91	71.81	2.7280
Y2	61.87	7.16	2082	0.1001	6.10	16.88	15.68	61.34	2.7821
CR1	72.35	8648	4905	0.1228	4.89	14.41	11.48	69.22	2.7616
CR2	58.38	7.16	2601	0.1099	6.51	17.27	13.56	62.66	2.7811
CT1	85.21	9627	4982	0.1128	7.71	4.70	10.11	77.48	2.6352
CT2	54.02	7.72	1052	0.0690	7.97	15.51	24.93	51.59	2.7897
T1	88.16	8924	2877	0.0862	5.45	6.26	19.26	69.03	2.6158
T2	61.95	7.15	2418	0.0870	6.78	16.21	15.75	61.26	2.7819
H	71.55	11,140	4914	0.1615	4.95	14.74	10.09	70.22	2.7302
J	53.02	13.67	2419	0.0884	8.14	14.37	12.78	64.71	2.7701
Z	116.70	10,720	7381	0.1248	3.45	7.05	11.78	77.72	2.6085
L	62.58	9848	2408	0.1090	6.50	13.10	17.60	62.80	2.7663
X	74.17	8446	4359	0.1106	6.01	8.55	13.47	71.97	2.6593
K0	68.79	10,750	6263	0.097	7.02	11.13	12.78	69.07	2.7229
K1	51.11	7.17	6662	0.0806	8.30	12.78	10.05	67.87	2.7327
K2	69.17	15,620	6120	0.1114	6.46	11.85	13.02	68.67	2.7223
K3	52.17	7.146	3955	0.068	8.97	10.88	16.47	63.68	2.7369

Regarding the effects of powder-liquid ratio and cement ratio, the total pore volume and various characteristic pore sizes of the joint sealants decrease continuously with the increase in powder-liquid ratio (Y1 → DZ → Y2). In particular, the pore size area inhabiting the most probable pore size of specimens in group Y2 shrinks from the macropore area to the gel pore area. Besides, the percentage of macropores decreases, the proportion of pores with diameters below 1000 nm increases, and the pore structure is refined overall. The main reasons are that at high powder-liquid ratios, the solid content volume inside the joint sealants is relatively large, and the materials have high density and strong resistance to dry shrinkage deformation. As a result, the pores formed after water evaporation are small in terms of their number and size, and the originally closed small pores are not easily inter-connected. The effects of cement ratio on the pore structure of joint sealants basically exhibit identical trends. With increase in cement ratio (CR1 → DZ → CR2), the total pore volume of joint sealants and various characteristic pore sizes decrease continuously, and the gel and transition pores show marked increases in quantity, whose pore size distribution moves gradually towards the micropore end. This suggests that increasing the cement ratio leads to decrease of pore volume, diminishing of pore size and enhancement of density inside the joint sealants. This phenomenon is primarily attributable to the increasingly pronounced water absorption and hydration effects of cement with its increasing dosage. On the one hand, this reduces the evaporation of water and the number of dry shrinkage pores resulting thereby. On the other hand, this leads to a continuous increase in cement hydrates. Owing to the filling and blocking effects of these hydrates, the pores inside the joint sealants can be reduced and refined accordingly.

Regarding the effects of cement type and filler blending, the hydration effect becomes weaker after changing to the low-grade white cement (DZ → CT1) because of the lower cement grade, so that less hydrates are produced and more amount of water is evaporated. Consequently, the total pore volume of joint sealants and various characteristic pore sizes all increase, and the pore structure is coarsened overall. After blending with sulphoaluminate cement (DZ → CT2), the joint sealants exhibit drastically decreased total pore volume and characteristic pore sizes, with the percentage of macropores being only 51.59%. This suggests remarkable refinement in the pore structure of joint sealants. The main reason is that the sulphoaluminate cement sets and hardens quickly upon encounter of water to generate substantial hydrates, which greatly reduces the evaporation of water and hinders the formation, communication and expansion of internal pores. After blending with talc powder (DZ → T1) or mica powder (DZ → T2), the total pore volume is reduced and the density is improved for the joint sealants. This is primarily owing to the accumulating and filling actions of the two fillers. Nevertheless, the sizes of various characteristic pores decrease after blending with mica powder, while showing slight increases after blending with talc powder. This is attributed to differences in the fineness and crystal morphology between the two fillers, which thus produce varying effects on the pore size distribution of the sealants. For instance, after blending with mica powder, the percentage of macropores in the joint sealants decreases, whereas the percentages of capillary, transition and gel pores increase. Contrastively, after blending with talc powder, the percentages of gel and transition pores decrease, whereas the percentages of macropores and capillary pores increase.

Regarding the effects of emulsion blending and latex powder addition, the total pore volume, various characteristic pore sizes and macropore percentage all increase significantly for the joint sealants after blending with styrene-acrylic emulsion (DZ → H). The total pore volume and most probable pore size, in particular, increase by 45.4% and 41.8%, respectively. This is primarily attributed to the relatively thin styrene-acrylic emulsion, which results in a small solid content volume, as well as substantial water evaporation. After adding latex powder (DZ → J), the content of polymer fraction in the joint sealants increases, and the total pore volume and various characteristic pore sizes somewhat decrease by the dispersive, film forming and filling actions of the latex powder.

With respect to the effects of additive and fiber addition, the total pore volume and various characteristic pore sizes all increase markedly after incorporating plasticizer (DZ → Z). The heightened percentage of macropores to 77.72% and the mere10.50% occupation of gel and transition pores suggest that the density of joint sealants is reduced, and the pore size distribution moves towards the macropore end. According to preliminary analysis, the probable reason is the thickening of newly mixed joint sealants by the swelling effect of plasticizer on emulsion particles, which makes the air bubbles not easily escapable and breakable. Meanwhile, with the absorption and evaporation of the plasticizer, the intermolecular force between some polymer molecules increases again. The process in which this internal stress tends to an equilibrium state again (similar to drying shrinkage) may also trigger production of certain amounts of pores. Further research is needed to clarify the specific cause of such phenomenon. Despite an increase in the most probable pore size after incorporating coupling agent (DZ → L), the total pore volume, mean pore size and median pore size of the joint sealants are all somewhat reduced. The change in pore size distribution is manifested by the decreased volume and percentage of macropores, as well as the increased volume and percentage of capillary pores. The reason is that the coupling agent enhances the interaction between inorganic and organic fractions in the joint sealants, thereby improving the material density. Meanwhile, the dispersion uniformity of inorganic powder is improved after surface modification with the coupling agent,which also lowers the probability of pore formation to a certain extent. After incorporating carbon fiber (DZ → X), the total pore volume of joint sealants remains fundamentally unchanged, while the sizes of various characteristic pores all increase to some extents, suggesting coarsening of the pore structure. The main reason is that the distribution density of the fibers inside joint sealants is not entirely uniform after carbon fiber blending. Thus, at sites with dense fiber distribution, the material cohesion and density are improved by the bridging and anti-cracking functions of the fibers, and the number of pores exhibits an overall decline. Contrastively, at sites with thinner fiber distribution, the material cohesion is relatively small, thereby resulting in a greater shrinkage deformation following film formation and hardening, as well as an increased volume of macropores.

As for the effects of various processing conditions, the polymer fraction in the joint sealants undergoes certain cross-linking under temperature aging after cold drawing and hot pressing. Besides, the hot pressing process leads to closing of partial pores inside the materials. Thus, despite less change in the median pore size of joint sealants than without treatment at this time, the total pore volume and mean pore size decrease markedly, especially the most probable pore size. Besides, the gel and transition pores increase slightly in terms of percentage, and their structures undergo certain degrees of refinement. After wet–dry cycling, part of the cement hydrates and inorganic components of the sealants are lost by the hydrolytic actions to form pores, thereby resulting in increased total pore volume and most probable pore size. Nevertheless, the median and mean pore sizes change little as compared to without treatment, and the percentages of various pore types remain fundamentally unchanged. This suggests that there is no obvious change in the pore size distribution of the sealants following wet–dry cycling. After long-term ultraviolet irradiation, the total pore volume and various characteristic pore sizes all decrease, and more macropores and transition pores are refined separately into capillary and gel pores. The primary reason is cross-linking of partial polymer molecules under ultraviolet irradiation aging, which improves the sealant density.

On the whole, for the polymer-cement composite joint sealants prepared in this paper, the internal pore structure is dominated by macropores with sizes above 1000 nm (almost accounting for over 60% of the total pore volume), while the volume of gel pores sizing below 10 nm is relatively small (accounting for less than 10% of the total pore volume). In addition, the total pore volume of joint sealants reflects the magnitude of internal total pore volume, which characterizes the overall material density. The variation trends of various characteristic pore sizes are overall consistent with the variation trend of total pore volume. This is because the improvement of material density is often accompanied by refinement of pore structure, and vice versa. Nevertheless, given the correlation of the magnitudes of various characteristic pore sizes with the pore size distribution, the pore size distribution changes greatly in some cases or the variation trends of total pore volume and pore size distribution are inconsistent. Hence, there is also a case where the individual characteristic pore size increases, or the overall pore structure is coarsened despite a decrease in total pore volume (e.g. the specimens in groups T1 and L, and those cold-drawn and hot-pressed specimens in group K of this section). Clearly, it is not advisable to infer the pore structure properties of joint sealants by change of a certain index. Instead, they should be determined through a multi-index comprehensive approach based on changes of multiple pore structure parameters^23,24^.

### Fractal characteristics of pore structure

This section describes the fractal characteristics of pore structure inside the studied joint sealants based on the fractal model of thermodynamic relationship^25^.With this model, the assumption on pore structure in the process of fractal dimension solving is closer to the actual situation. The essential basis for the model is that the increase in surface energy of mercury liquid during the MIP process is equal to the work done by the external force on the mercury. In other words, the following relationship is established between the pressure on mercury $p_{{\text{h}}}$ and the mercury intrusion volume *V*_h_^25,26^:

1 $$\int_0^{{V_h}} {{p_{\rm{h}}}{\rm{d}}V = - \int_0^S {{\sigma _{\rm{h}}}\cos \theta {\rm{d}}S} }$$

where ${{\sigma _{\rm{h}}}}$ denotes the surface tension of mercury, $\theta$ denotes the contact angle between mercury and specimen, and *S* denotes the pore surface area of specimen.

Through dimensional analysis and discretization of Eq. (1), the measured pressure and mercury intrusion volume can be correlated with the surface fractal dimension of the material pore structure. Accordingly, the fractal model expression is derived as follows:

2 $$\sum\limits_{i = 1}^n {{{\bar p}_h,i}\Delta {V_{h,i}} = C'd_n^2{{(V_{h,n}^{1/3}/{d_n})}^{{D_{\text{p}}}}}}$$

where *n* denotes the number of pressure application intervals during mercury intrusio, ${\bar p_{h,i}}$ and $\Delta {V_{h,i}}$ represent the mean pressure and intrusion volume during the *i-*th mercury intrusion, $C'$ is a constant obtained from fitting, which has no specific physical meaning, ${d_n}$ and $\Delta {V_{h,n}}$ represent the pore size and cumulative intrusion volume corresponding to the *n*th mercury intrusion, and ${{D_{\text{p}}}}$ is the fractal dimension of pore surface area that is calculated based on the thermodynamic relationship (hereinafter referred to as the fractal dimension). Let ${W_n} = \sum\limits_{i = 1}^n {{{\bar p}_{h,i}}\Delta {V_{h,i}}}$ and ${Q_n} = V_{{\text{h}},{\text{n}}}^{1/3}/{d_{{\text{h}},{\text{n}}}}$, then the Eq. (2) can be further rewritten as:

3 $$\ln \left( {{W_n}/d_n^2} \right) = {D_p}\ln {Q_n} + \ln C'$$

According to Eq. (3), $\ln \left( {{W_n}/d_n^2} \right)$ and $\ln {Q_n}$ can be directly solved by utilizing the MIP measurement data. The slope of the linearly fit line between the two is denoted by ${{D_{\text{p}}}}$.

**Figure 13:**
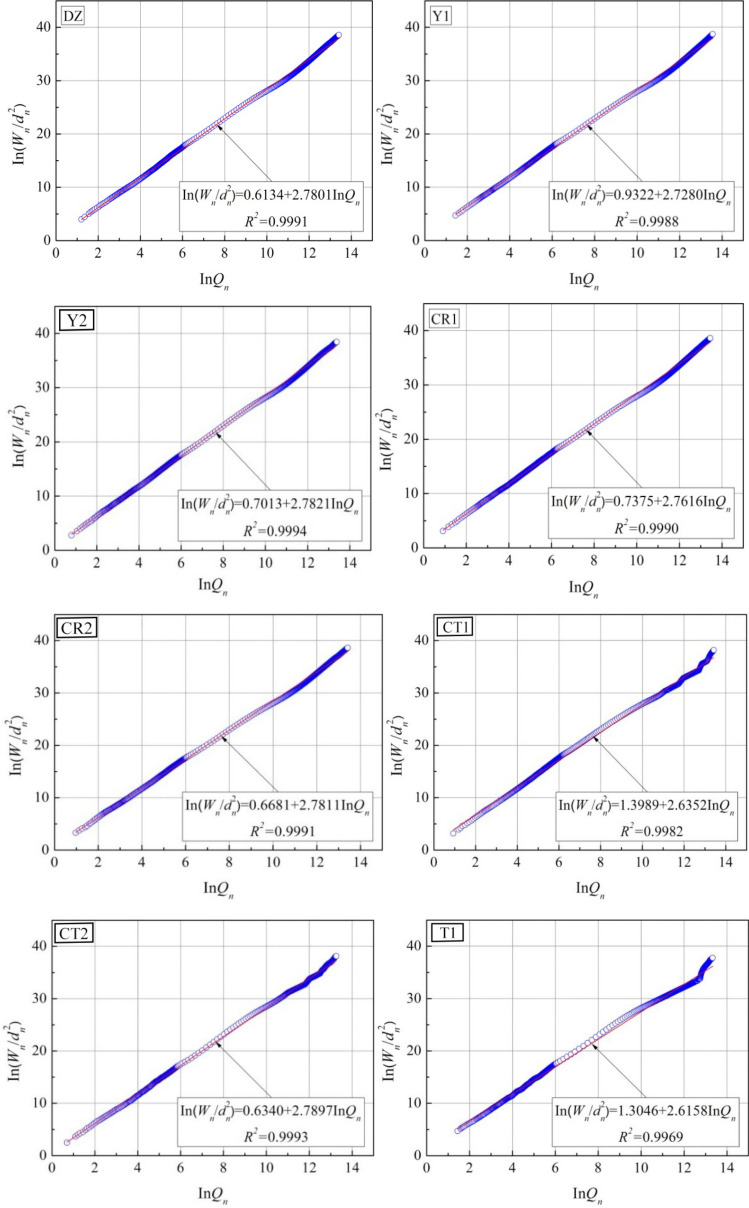

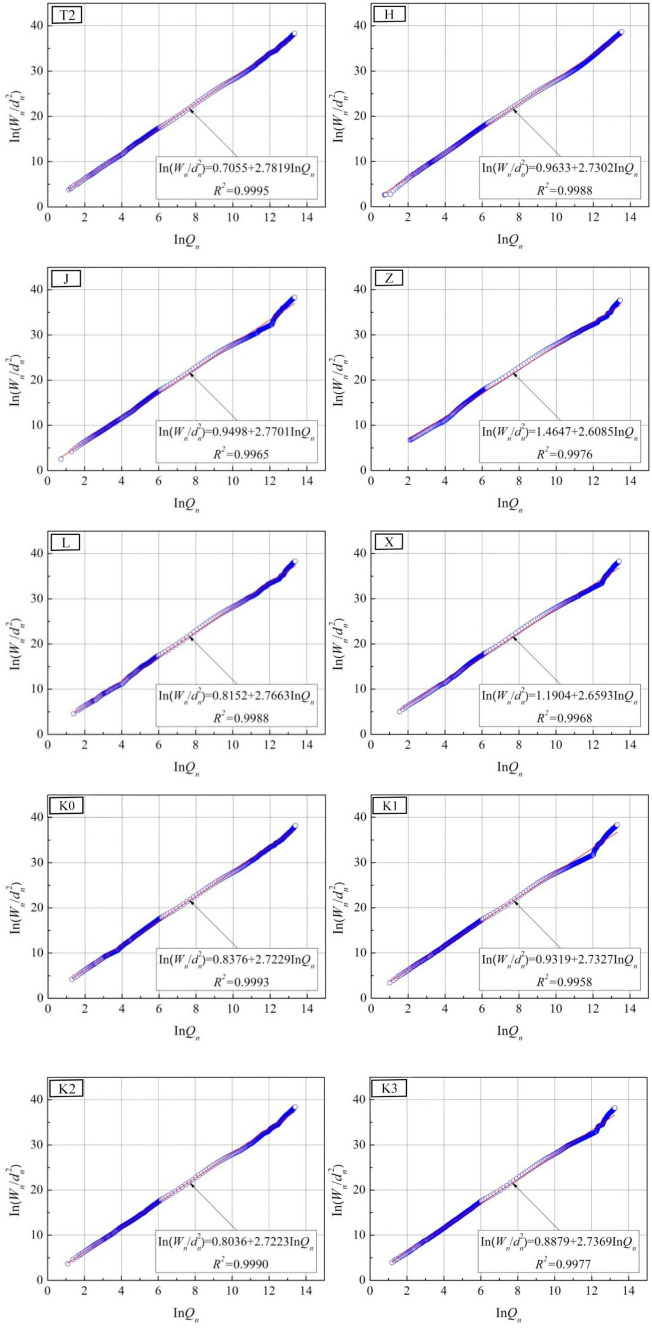
$\ln (W_{n} /d_{n}^{2} )-\ln Q_{n}$ scatter plots and fitted lines.

**Figure 14:**
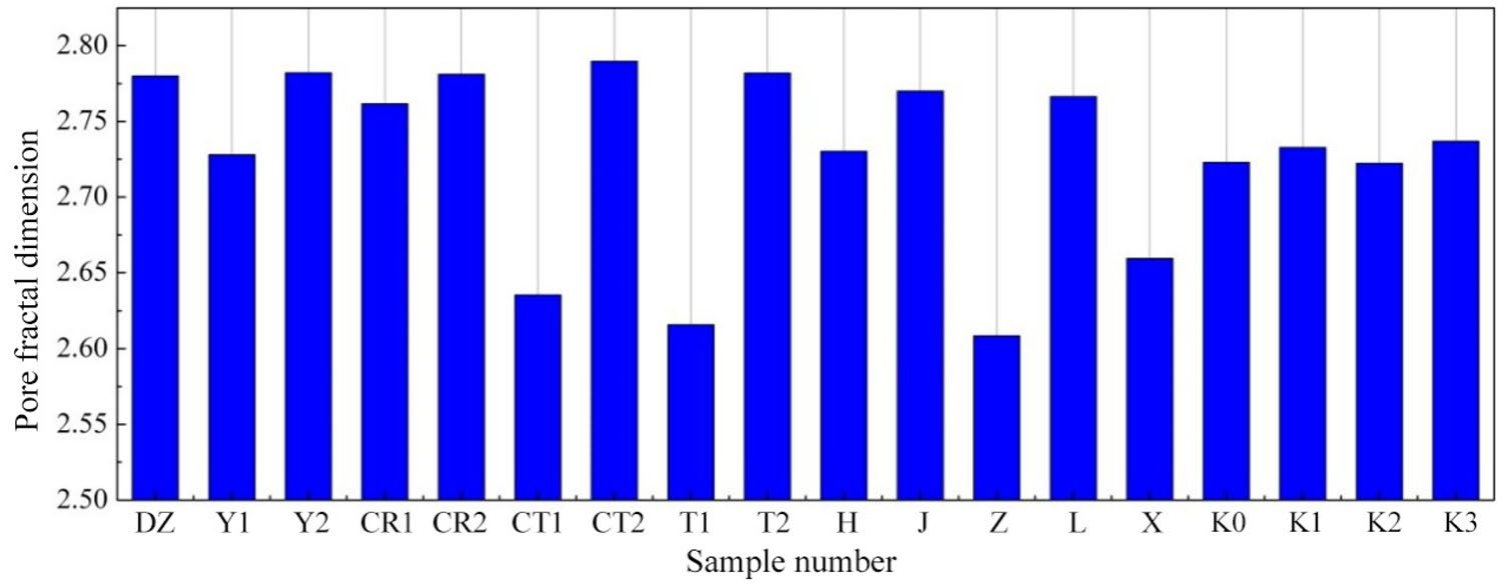
Fractal dimension of pore surface area.

In Fig. 13, the $\ln \left( {{W_n}/d_n^2} \right)-ln {Q_n}$ scatter plots of tested specimens in various groups are presented, as well as their fitted lines. Figure 14 displays the corresponding computational results for fractal dimension. As is clear, the correlation coefficients (*R*^2^) of the fitted lines for specimens in various groups are all greater than 0.99, suggesting distinct fractal characteristics of the pore structure for the studied joint sealants. Within the range of raw material ratios involved in this section, the fractal dimension values for the pore surface area of the sealants are between 2.6 and 2.8.

Since the fractal dimension characterizes the overall disorderliness and complexity of sealant pore structure, larger fractal dimension signifies more irregular and more complex pattern of the sealant pore size distribution. With respect to the MIP results, such complexity enhancement is often manifested as the refinement of pore structure. In other words, the percentage of small-sized pores increases, while the percentage of large-sized pores decreases. Conversely, when the pore size distribution of the joint sealants moves towards the macropore end, the fractal dimension value shows a corresponding decrease. For instance, as suggested in the foregoing analysis of basic pore structure parameters, when the powder-liquid ratio of the sealants increases from 0.30 to 0.55 (Y1 → Y2), various pore structure parameters all decrease, the percentage of macropores drops from 71.81 to 61.34%, and the corresponding fractal dimension value increases from 2.7280 to 2.7821. After changing to low-grade white cement (DZ → CT1), or blending with styrene-acrylic emulsion (DZ → H), the pore structure parameters of joint sealants all increase to varying degrees. The percentage of macropores increases by 13.40% and 6.13%, the percentage sums of gel and transition pores decrease by 9.19% and 1.91%, and the corresponding fraction dimension values​are reduced to 2.6352 and 2.7302, respectively. The pore structure inside the joint sealants is refined overall after long-term ultraviolet irradiation treatment,and the fractal dimension value increases from 2.7229 (without treatment) to 2.7369. After the wet–dry cycling treatment, little changes are observed regarding the percentages of pores within various size ranges, despite the increase of total pore volume inside the sealants. This indicates that the pattern of pore size distribution remains basically unchanged, so the corresponding change in fractal dimension value is also small (2.7223).

**Figure 15:**
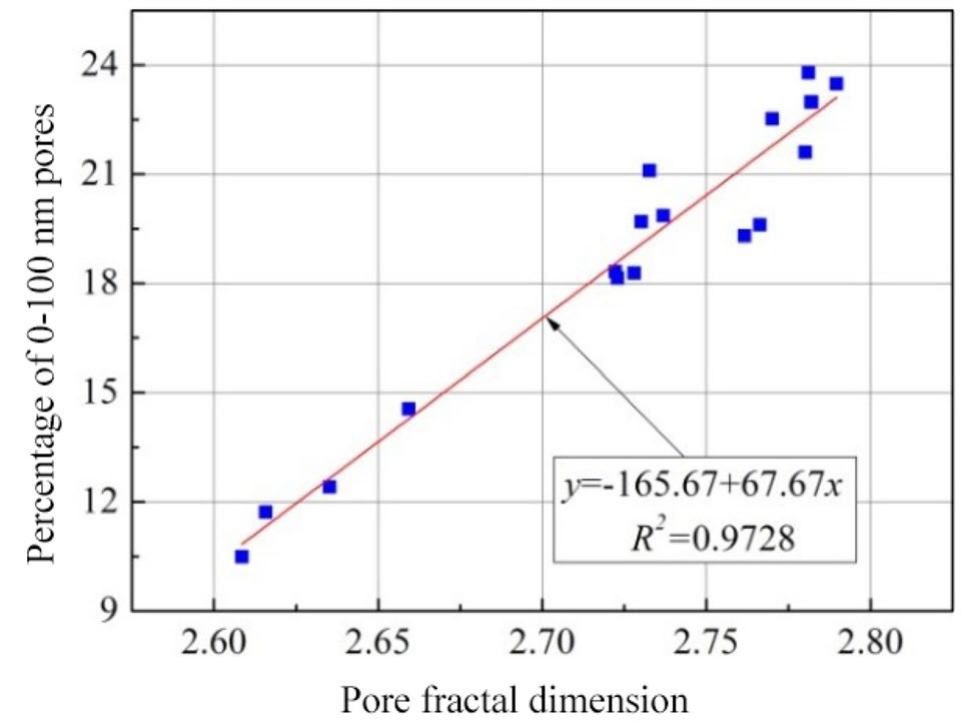
Changes in the percentage of 0–100 nm pores with pore fractal dimension.

**Figure 16:**
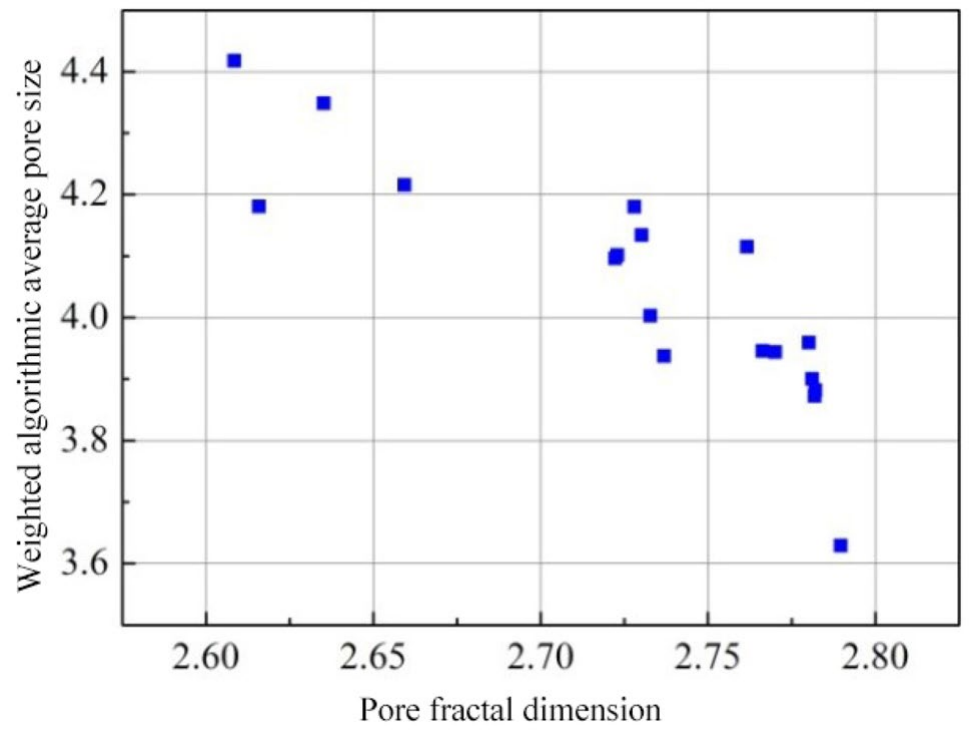
Changes in weighted algorithmic average pore size with pore fractal dimension.

To further verify the above assumption, Fig. 15 lists the relationships between the pore fractal dimension value of each specimen and the percentage of 0–100 nm pores (namely gel pores and transition pores). Meanwhile, the weighted logarithmic average pore size *δ* is defined as:

4 $$\delta = \sum\limits_g {{\lambda _g}\lg ({d_g})}$$

where the subscript *g*represents the four types of pore size ranges mentioned above, including macropores, capillary pores, transition pores and gel pores, $\lambda_{g}$ stands for the percentage of each type of pores in the total pore volume, i.e. weight, $d_{g}$ is the representative pore size of each type of pores, i.e. the average of upper and lower limits of corresponding pore size ranges.The upper limit of macropores is the maximum pore size measured during the test. Figure 16 presents the correlations of pore fractal dimension value of each specimen with the weighted logarithmic average pore size.

It is clearly observed from Figs. 15 and 16 that, on the whole, the proportion of small-size (0–100 nm) pores in the joint sealants increases with the increasing pore fractal dimension, whereas the weighted logarithmic average pore size decreases in a gradual manner. Besides, a good linearity is observed between the pore fractal dimension of joint sealants and the percentage of 0–100 nm pores. Thus, clearly, the fractal dimension of pore surface area obtained in this study is an ideal characterization of the complex pore structure of polymer-cement composite joint sealants for pavements. In addition, changes in the relative contents of pores with different sizes in the joint sealants may be effectively described by the fractal dimension value.

## Conclusions

In this paper, the basic microstructure and pore structure properties of polymer-cement composite joint sealants are studied through SEM observation and MIP experiment, and the effects and rules of various material types, ratio parameters and processing conditions on the sealant microstructure and pore structure parameters are analyzed. The following conclusions are drawn:

1. A basic microstructure is formed inside each polymer-cement composite joint sealant, where the polymer film serves as a continuous base phase, and various inorganic components and cement hydrates are embedded and wrapped within the polymer base phase as a dispersed phase. Besides, an interpenetrating network structure is also present locally, which is formed by interweaving of the polymer film with the inorganic fillers and cement hydrates.
2. Regarding the internal pore structure of the studied polymer-cement joint sealants for pavements, the macropores with sizes above 1000 nm are predominant. Pore size distribution presents distinct fractal characteristics, and the corresponding fractal dimension of pore surface area ranges between 2.6 and 2.8. Greater value of fractal dimension indicates higher complexity of pore size distribution pattern for the joint sealants. In other words, with the increasing proportion of micropores, the proportion of macropores decreases, and the pore structure undergoes an overall refinement.
3. Changes in raw material type, ratio parameter and processing condition produce insignificant effects on the basic microstructure properties and configuration of joint sealants, with effects reflected primarily in the change of sealant pore structure. Measures for reducing the total pore volume of joint sealants and refining their pore structure include enlargement of powder-liquid ratio, incorporation of sulphoaluminate cement or mica powder, addition of latex powder, etc. Contrastively, changing to low-grade cement, incorporating styrene-acrylic emulsion or adding plasticizer yields the opposite effects.
4. This article only analyzes the microstructure and pore structure of the polymer-cement composite joint sealants for pavements at the microscopic level. In the next step, further quantitative analysis is needed concerning changes in the composition of various materials inside the joint sealants at different ratios and conditions by employing other microscopic test methods, as well as the chemical reaction mechanisms between organic and inorganic components.
